# Phase II study of ‘high-dose’ celecoxib and metronomic ‘low-dose’ cyclophosphamide and methotrexate in patients with relapsed and refractory lymphoma

**DOI:** 10.3332/ecancer.2009.144

**Published:** 2009-08-05

**Authors:** N Abdel-Bary, T Hashem, H Metwali, A Abd el Ghany, HA Magied, M El-Herbeiny

**Affiliations:** Department of Clinical Oncology and Nuclear Medicine, Faculty of Medicine, Menoufia University, Egypt

## Abstract

**Introduction:**

Relapsed histologically aggressive non-Hodgkin’s lymphoma (NHL) has a poor prognosis; relapsed patients who respond to second-line chemotherapy have a better outcome after BMT, while those who do not respond to second line or are unfit for BMT have a worse prognosis and new treatments are needed. Angiogenesis is increased in aggressive NHL and could be targeted by selective cyclooxygenase-2 inhibition and metronomic chemotherapy.

**Aim of the study:**

Assessment of the toxicity of metronomic chemotherapy and the response and progression-free survival of relapsed diffuse large B cell lymphoma (DLCBL) patients.

**Patients and methods:**

Forty patients with the diagnosis of relapsed and/or refractory DLCBL. Patients included in this study may have received any number of preceding therapies (as long as one had included an anthracycline) and were not candidates for BMT. They received cyclophosphamide tab (50 mg p.o. q.d), methotrexate tab (2.5 mg p.o four times per week) and high-dose celecoxib tab (400 mg p.o. b.i.d.) until disease progression or toxicity.

**Results:**

All of the 40 patients included (median age, 56 years) were evaluable for response, 52% had a high international prognostic index at relapse, with a median follow-up of 8.4 months (range 4–23 months), 32.5% had a partial response and 50% has stable disease. Progression-free survival was 12 months. The median response duration was nine months. Treatment protocol was well tolerated with no major toxicities. The most common toxicity was fatigue (57.5%), myelo-suppression and gastrointestinal side effects.

**Conclusions:**

Low-dose cyclophosphamide, methotrexate and high-dose celecoxib are well tolerated and active in pre-treated diffuse large cell B lymphoma. Although thrombotic events were not observed during this study, close surveillance for arterial and venous thrombotic events is recommended.

## Introduction

Diffuse large B cell lymphoma (DLBCL), the most common subtype of non-Hodgkin lymphoma (NHL), is an aggressive disease with considerable biologic and clinical heterogeneity. Standard first-line treatment is combination chemotherapy with cyclophosphamide, doxorubicin, vincristine and prednisone (CHOP) or an equivalent regimen combined with rituximab (R). This results in a complete remission rate of 75–80% and a 3–5-year progression-free survival (PFS) of 50–80%. The addition of R to CHOP chemotherapy has been the most significant step in recent years in improving overall survival (OS) and PFS rates. Current treatment with CHOP-R results in an improvement in PFS and OS by 15–20% compared to CHOP chemotherapy alone [1].

Despite this major therapeutic advance, a significant proportion of patients will relapse or remain refractory to initial chemo-immunotherapy. The PARMA trial confirmed the place of high-dose chemotherapy and autologous haematopoietic cell transplantation (aHCT) as the optimum salvage treatment. In this trial, chemotherapy-sensitive patients were randomized to further salvage chemotherapy with cytarabine/platinum-based chemotherapy alone, or in combination with aHCT. Event-free survival and OS at five years in the transplant arm were 46% and 53%, respectively, compared with 12% and 32% in the chemotherapy alone arm [2].

Numerous salvage chemotherapy regimens have been used to treat relapsed or refractory DLBCL patients. The majority are based on agents that demonstrate non-cross-resistance to those used in primary therapy. Broadly speaking, they can be divided into regimens based on ifosfamide, cytarabine/platinum or gemcitabine. Studies on salvage therapy have generally included all patients with aggressive lymphoma and are not restricted to DLBCL subtype [3].

Recent data indicate that angiogenesis is important in the pathophysiology and prognosis of aggressive histological subtypes of NHL. Angiogenesis is a multi-step process that leads to the formation of new blood vessels from existing vasculature and is associated with the growth and dissemination of malignant tumours [4]. Angiogenesis and angiogenic factors are increased in most lymphomas. In addition, angiogenesis has been associated with adverse outcome or more aggressive clinical behaviour in malignant lymphoma. However, the role of angiogenesis might vary in lymphoma subtypes because of the prognostic value of micro-vessel density and the different expression of angiogenesis-related molecules in the various lymphoma subtypes [5].

Metronomic chemotherapy refers to the frequent, even daily, administration of chemotherapeutics at doses significantly below the maximum tolerated dose, with no prolonged drug-free breaks [6].

Much evidence, mostly ***in vitro***, indicates that the ‘activated’ endothelial cells of newly forming blood-vessel capillaries are highly and selectively sensitive to very low doses of various chemotherapeutic drugs [7].

The interesting new findings of Wun *et al* [8] reveal that human Burkitt-type B-cell lymphoma cell lines express elevated levels of Cyclooxygenase 2 (COX-2), and this fits with earlier studies indicating that B-lineage cells are capable of expressing COX-2. COX-2 is a prostaglandin synthase enzyme that has been implicated in tumourigenesis. One of the proposed mechanisms by which this may occur is by stimulating angiogenesis through the production of pro-angiogenic factors, including VEGF, basic fibroblast growth factor, platelet-derived growth factor, transforming growth factor-h1 and endothelin-1. COX-2 inhibitors, such as celecoxib, have been shown to reduce the incidence of neoplastic lesions in familial adenomatous polyposis and therefore may have a role in the treatment of established malignancies. Furthermore, COX-2 is over-expressed in some lymphomas and is of potential prognostic importance. These pre-clinical data provide the rationale for using low-dose chemotherapy together with a selective COX-2 inhibitor in the treatment of aggressive NHL [9].

## Patients and methods

From March 2006 to June 2008, 40 patients with relapsed or refractory diffuse large cell lymphoma were enrolled in this phase II study. The primary objective was to determine the response rate to oral high-dose celecoxib combined with daily oral low-dose cyclophosphamide and low-dose methotrexate continuous administration in this patient population. The secondary objectives were to define the toxicity of the given regimen and PFS.

Patients were considered eligible if they had relapsed after any number of preceding therapies (as long as one had included an anthracycline) and had a projected life expectancy of >4 months.

Patients who relapsed following autologous haematopoietic stem cell transplant were eligible. Patients were excluded from the study if they were transplant eligible, receiving concurrent chemotheraphy or radiotherapy (including corticosteroids) or had received any other antineoplastic therapy within the preceding two weeks. Patients with Eastern Cooperative Oncology Group performance status of >3, uncontrolled hypertension, and unstable cardiovascular or significant renal or hepatic disease were also excluded. As celecoxib is a sulphonamide, patients with a proven allergy to sulfa drugs were also excluded.

Adequate haematological (haemoglobin count, >85 g/l; absolute neutrophil count, >1000/mm^3^; platelet count, >75,000/mm^3^), renal, and hepatic functions were mandatory. All eligible patients had at least one bi-dimensionally measurable target lesion. Our institutional review board approved this study and informed consent was obtained from each patient.

Pre-treatment evaluations included a complete history and physical examination, routine laboratory evaluation, and computed tomography of chest, abdomen and pelvis as well as bone marrow biopsy. Patients were clinically assessed by physical examination and blood work monthly until progression. Computed tomography scans were repeated for response evaluation every two months or sooner if clinically indicated. Patients were followed off-study until death.

## Treatment plan

All patients received celecoxib 400 mg p.o. b.i.d. (with food) and cyclophosphamide 50 mg p.o. daily, methotrexate 2.5 mg p.o four times per week. In cases of nausea and vomiting (> grade 3), dyspepsia or abdominal pain or a 50% increase in serum creatinine or liver enzymes celecoxib was reduced (200 mg p.o. b.i.d. then 100 mg p.o. bid). The need for further dose reduction or gastrointestinal bleeding resulted in discontinuation within the study. Similarly, cyclophosphamide could be reduced twice (to 25 mg p.o. daily then 25 mg p.o. alternate days) for grade >3 neutropenia or thrombocytopenia. No dose escalations were permitted.

Toxicity was assessed according to Common Terminology Criteria for Adverse Event v3.0, published 12 December 2003.

## Response assessment

Response to treatment was assessed according to criteria of ***Cheson et al*** [10].

An objective response consisted of complete clinical response, complete clinical response unconfirmed or a partial response. Progression of disease was determined by an increase of 50% in the product of perpendicular diameters of the index lesions or the appearance of new lesions. All other patients were considered to have stable disease.

Because anti-angiogenic therapy is cytostatic, and in many pre-clinical models of metronomic chemotherapy, tumours may initially grow before they stabilize and sometimes regress, patients were allowed to remain in the study for up to four months if radiological or clinical tumour progression was asymptomatic and not a threat to vital organ function.

## Statistical analysis

Data were analysed using the SPSS programme for Windows version 11. PFS was estimated using the Kaplan-Meier estimate. Results were considered significant at the 5% critical level (p-value < 0.05)

## Results

This prospective study included 40 patients with refractory or relapsed DLBCL who presented at the Menoufia University Hospital, Clinical Oncology Department between March 2006 and June 2008 and were followed up until the end of December 2008. All patients had measurable disease. Patients received low-dose oral cyclophosphamide tab 50 mg daily, methotrexate 2.5 mg tab four times per week and high-dose celecoxib tab 400 mg p.o bid.

## Patient characteristics

- **Initial pathologic status:** all patients had pathologically diagnosed DLBCL.

**Age and sex distribution:** the median age was 56 years (range 38–78) with equal male to female ratio (20 male and 20 female) (see Table 1)

- **IPI status:** At relapse, 21 patients had a high intermediate IPI score (52.5%), six patients (15%) had a low intermediate score, three patients (7.5%) had a low score, while ten patients (25%) had a high score.
- **Number of prior chemotherapy regimens** only 18 patients (45%) received this protocol for relapsed DLCBL after failure to achieve complete remission with CHOP or relapsed after completing their CHOP course, while nine patients (22.5%) had received one line of chemotherapy for relapsed disease, 11 patients (27.5%) had received two lines of treatment and two patients (5%) received three lines of treatment for relapse. Time from last relapse ranged from one1 to ten months with a median of 1.5 months. For patients who received one line of treatment for relapse the time from last relapse ranged from three to ten months with a median of eight months. For patients who received two lines of treatment for relapse the time from last relapse ranged from one to nine months with a median of three months. For the two patients who received three lines, the time from the first relapse was one and three months, respectively. Here was significant correlation between the number of prior protocols and the time to relapse; patients who received only one line for relapse had a longer time to relapse (p = 0.05). Also, there was significant correlation between PFS and age of patients (by Kendall’s tau test). The number of prior chemotherapy protocols given for relapse did not (statistically) affect PFS in our study.

## Toxicity assessment

**Figure 1:**
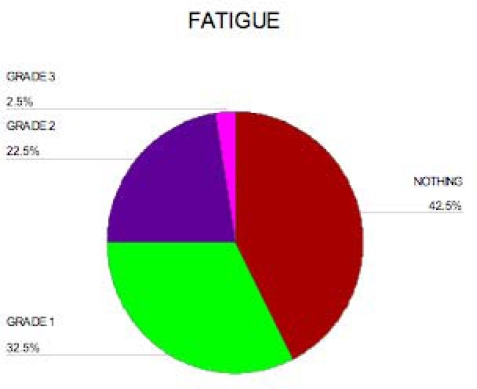
Percentage of fatigue among patients.

Toxicity was evaluated according to the National Cancer Institute’s Common Terminology Criteria for Adverse Events version 3.0. Fatigue was the most important side effect noticed in this study(see Figure 1), 13 patients (32.5%) had grade I fatigue, nine patients (22.5%) had grade II fatigue, while one patient (2.5%) had grade III fatigue. No dose reduction or interruption to treatment was necessary except for the patient with grade III for whom treatment was interrupted for one week then resumed at the usual dose.

Gastrointestinal toxicities were mild ranging from grade I to grade II, with no grade III or IV toxicities observed, except for two patients who had grade III oral mucositis. Both these patients had undergone prior whole neck irradiation for treatment of cervical lymph nodes; metronomic chemotherapy had to be stopped for two weeks until the condition improved.

Six patients experienced grade I nausea (15%), two patients had grade II nausea (5%), while only five patients had grade I vomiting (12.5%). Dyspepsia was observed in ten patients, eight patients (20%) had grade I dyspepsia, while two patients (5%) had grade II dyspepsia. Grade I oral mucosities was observed in eight patients (20%).

## Haematological toxicity

Anaemia was observed in eight patients, six (15%) had grade I anaemia while two (5%) had grade II anaemia. Grade I thrombocytopenia was observed in four patients (10%). Neutropenia was observed in seven patients, six patients had grade I neutropenia (15%), while one patient (2.5%) had grade II neutropenia. No dose reduction was needed for haematological toxicities.

Three patients (7.5%) had grade I skin rash, which responded to topical corticosteroid and oral antihistamines.

Three patients had grade I elevated serum creatinine (>ULN - >1.5 × ULN) and no dose reduction of celecoxib was necessary. One patient had grade II elevated serum creatinine (1.5 - 3 × ULN) and the celecoxib dose was reduced to 200 mg b.i.d.

Two patients had grade I oedema, which responded to a short course of diuretics with no treatment interruption.

There was no hepatic toxicity reported in this study as regards elevated liver enzymes or elevated bilirubin levels.

## Response rates

Thirteen patients (32.5%) had a partial response; no complete responses were achieved in this study. Twenty patients (50%) had stable disease. Seven patients (17.5%) progressed, four of whom died due to progressive disease while the other three were shifted to another line of chemotherapy. For the whole study population, time from diagnosis of initial disease (i.e. primary diagnosis of lymphoma—before relapse or resistance to CHOP) until last follow-up ranged from five to 85 months with a median of 18 months.

Among the patients with a partial response, four ultimately progressed while on therapy at a median of nine months, with a range of 6–12 months. Eight patients with stable disease developed a progressive course at a median of 8.5 months with a range of 5–23 months.

Patients who progressed were shifted to another line of treatment or kept on supportive care depending on their general condition.

## Progression-free survival

**Figure 2:**
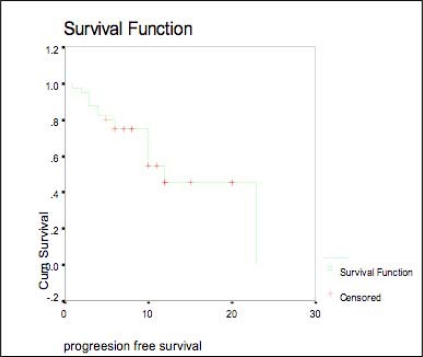
Progression-free survival.

The median actuarial PFS was 12 months (95% CI, 7.85–16.15), (see Figure 2)

There was a significant correlation between PFS and age (p-value 0.04).

There was no significant correlation between PFS and time from last relapse, number of prior lines of chemotherapy or time from last treatment.

Also, there was no significant correlation between IPI score and PFS (p = 0.07).

## Discussion

Targeting angiogenesis has also been investigated in the Southwest Oncology Group S0108 study of the anti-VEGF monoclonal antibody, bevacizumab, in patients with relapsed DLBCL. A clinical non-progression rate of 25% was observed for the 51 patients in this study, with a median time to progression of five months (range 4–18 months) [11].

In our study, we have shown that the combination of continuous low-dose cyclophosphamide, methotrexate with high-dose celecoxib resulted in a response rate of 32.5% in a group of pre-treated patients with relapsed DLBCL. The combination was well tolerated, with a low incidence of haematological, gastrointestinal and renal toxicity.

There is only one published study regarding the use of metronomic chemotherapy in treatment of relapsed NHL, by Buckstein *et al* [9] in which 32 patients with relapsed or refractory NHL were enrolled to receive high-dose celecoxib 400 mg b.i.d daily and low-dose cyclophosphamide 50 mg p.o daily. Only 20 patients had relapsed or refractory DLBCL, others had anaplastic lymphoma, T-cell lymphoma or transformed follicular lymphoma. Most patients were heavily pre-treated, with a median of three prior chemotherapy regimens (range 1–7). Eleven patients (34%) had relapsed after autologous haematopoietic stem cell transplant.

In our study, we added methotrexate tab 2.5 mg four times per week, all patients had relapsed or refractory DLBCL with no other pathological types, in addition none of our patients had received aHCT.

None of our patients achieved complete remission while (32.5%) and (50%) achieved partial response and stable disease, respectively.

In our study, the median actuarial PFS was 12 months (95% CI, 7.85–16.15) compared to the study of Buckstein *et al* [9] in which the median actuarial PFS was 4.7 months (95% CI, 2.5–9.2).

In our study, only three patients had grade I skin rash, which developed months after initiation of treatment and responded to anti-allergic treatments; no interruption of the programme was needed. Fatigue was a common side effect; 13 patients (32.5%) had grade I fatigue, nine patients (22.5%) had grade II fatigue, while one patient (2.5%) had grade III fatigue. No thrombotic events were observed in our study.

## Conclusion

We conclude that metronomic chemotherapy can be used for patients with relapsed and or refractory DLBCL with reasonable outcome and acceptable toxicity. Standard lines such as HST and chemo-immunotherapy combinations should be explored prior to a decision on metronomic chemotherapy.

## Recommendation

A longer follow up is recommended to evaluate the late toxicities of metronomic chemotherapy schedule. Although there were no thromboembolic manifestations recorded in this study, it is important to closely monitor the patients for these events as they are exposed to high levels of celecoxib.

We suggest a study involving a larger number of patients and to correlate clinical response with VEGF blood levels and also to determine COX-2 receptor status before and during treatment to assess treatment benefit.

## Figures and Tables

**Table 1:**
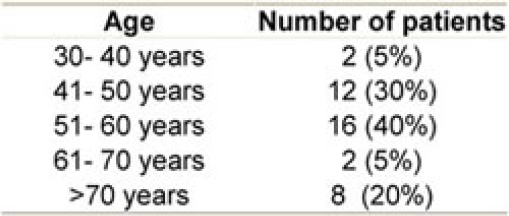
Age distribution among patients
